# Implementing a 6-month Hybrid Exercise Intervention for Older Adults: Feasibility of the SAY Exercise Trial

**DOI:** 10.1093/geroni/igaf122.2741

**Published:** 2025-12-31

**Authors:** Stephanie Voss, Shayna Basu, Nandini Erodula, Emily Erlenbach, Edward McAuley, Neha Gothe

**Affiliations:** Mass General Brigham, Boston, Massachusetts, United States; Northeastern University, Boston, Massachusetts, United States; Northeastern University, Boston, Massachusetts, United States; EXOS, South San Francisco, California, United States; University of Illinois Urbana-Champaign, Urbana, Illinois, United States; Northeastern University, Boston, Massachusetts, United States

## Abstract

Hybrid programs have become ubiquitous in the post pandemic world and could help overcome challenges faced by older adults (OA)s’ to engage in exercise. We present data on the feasibility, safety, and acceptability of a hybrid (in-person and virtual) yoga, aerobic and stretching exercise program for OAs. N = 145 (36 male, Mage= 64) low active OAs participated 3x/week for 6-months in a hybrid exercise program as part of a randomized controlled trial examining exercise effects on neurocognition. Attendance, attrition, format preferences, and adverse events were documented. Participants also completed a survey to detail their experiences and preferences with the hybrid delivery model. N = 119 completed the program with attendance ranging from 74% to 79%. On average across the three exercise groups, 37% and 63% of the attended sessions were on Zoom and in-person, respectively. On post-program surveys, a majority reported a preference for hybrid (50.4%) programs in the future, with only 19% reporting a preference for a fully virtual exercise program. Nine adverse events were reported, with 3 related or potentially related to the intervention and related assessments. Collectively, these findings demonstrate that a hybrid exercise program including modalities like yoga are feasible and safe for OAs, with participants attending 1 in every 3 sessions virtually. While elements of in-person exercise programming such as socialization with peers and hands on instruction with the exercise and yoga teachers were valued, the hybrid modality allowed OAs to overcome unique barriers which are paramount for adherence to exercise programs as well as other health interventions.

